# Association of interleukin 21 receptor gene variants with autoimune diseases in a type 1 diabetes cohort

**DOI:** 10.1186/1758-5996-7-S1-A219

**Published:** 2015-11-11

**Authors:** Cintia Semzezem, Marcia RS Correia, Aritania S Santos, Rosa T Fukui, Karla FB Gomes, Rodolfo Batista, Maria Elizabeth Rossi da Silva

**Affiliations:** 1 Faculdade De Medicina Da Universidade De São Paulo, São Paulo, Brazil

## Background

Type 1 Diabetes (T1D) is an autoimmune disorder mediated by T lymphocytes and dendritic cells. The lymphocyte activation involves the inflammatory pathways T helper1 (Th1), Th2 and Th17 and the inhibition of regulatory T cells. It was found that Th17 pathway is implicated in the inflammatory process termed insulitis, resulting in the destruction of pancreatic beta cells, being regulated by the interleukins IL-21, IL-23 and IL-27. Studies have demonstrated a role for the interation of IL21 and its receptor IL21R in the genesis and progression of many autoimmune diseases. The variant rs2214537 was associated with multiple sclerosis and Kawasaki disease and rs2285452 with thyroid disease.

## Objective

The aim of this study was to evaluate the influence of the variants rs2214537 and rs2285452 of the gene of IL21R (cr. 16p11) in susceptibility to T1D and the frequency of pancreatic and extra-pancreatic autoantibodies in patients with T1D in São Paulo.

## Materials and methods

We evaluated 631 patients with T1D (25.1±12.7) and 652 controls (28.6±11.4). The variants rs2214537 e rs2285452 were genotyped by the Vera Code Golden Gate (Illumina) methodology. Autoantibodies against zinc transporter 8 (anti-ZnT8) were determined by ELISA and anti-glutamic acid decarboxylase (anti-GAD65), anti-tyrosine phosphatase (anti-IA2), anti-thyroid peroxidase (anti-TPO), and anti-tireoglobulin (anti-TG) autoantibodies were measured by radioimmunoassay. Antinuclear (FAN) and anti-parietal cell (PCA) antibodies were measured by indirect immunofluorescence; rheumatoid factor by nephelometry and anti-TSH receptor antibodies (TRAb) by radioreceptor assay. The genotypic associations were analyzed using the Chi-square test or Fisher exact test.

## Results

The genotype frequencies of rs2214537 and rs2285452 variants were in Hardy-Weinberg Equilibrium, were similar in patients and controls and independent of gender. However, the CC genotype of the rs2214537 was associated with higher frequency of PCA (16.7% x 4.8%; p=0.0016, OR=3.93; CI=1.6-9.7). The AA genotype of rs2285452 increased the frequency of the anti-TPO (48.4% x 27.3%; p=0.01; OR=2.49; CI=1.19 to 5.24) and PCA (19.2 x 7.5%; p=0.04; OR=2.95; CI=0.99 to 8.75) p<0.05. There was no influence of these variants on the frequency of the other autoantibodies analyzed.

## Conclusions

The CC genotype of the variant rs2214537 and the AA genotype of the rs2285452 were related to higher frequency of extra-pancreatic autoantibodies, confirming their role in autoimmunity in T1D patients.

